# Induced degeneration and regeneration in aged muscle reduce tubular aggregates but not muscle function

**DOI:** 10.3389/fneur.2024.1325222

**Published:** 2024-01-26

**Authors:** Felipe Tadeu Galante Rocha de Vasconcelos, Antonio Fernando Ribeiro Júnior, Brandow Willy Souza, Isabela de Aquino Zogbi, Laura Machado Lara Carvalho, Letícia Nogueira Feitosa, Lucas Santos Souza, Nathália Gagliardi Saldys, Merari de Fátima Ramires Ferrari, Mariz Vainzof

**Affiliations:** Human Genome and Stem Cell Research Center, Department of Genetics and Evolutionary Biology, Biosciences Institute, University of São Paulo, São Paulo, Brazil

**Keywords:** tubular aggregates, muscle regeneration, aging, muscular dystrophies, neuromuscular disease

## Abstract

**Introduction:**

Tubular aggregates (TA) are skeletal muscle structures that arise from the progressive accumulation of sarcoplasmic reticulum proteins. Cytoplasmic aggregates in muscle fibers have already been observed in mice and humans, mainly during aging and muscle disease processes. However, the effects of muscle regeneration on TA formation have not yet been reported. This study aimed to investigate the relationship between degeneration/regeneration and TA in aged murine models. We investigated the presence and quantity of TA in old males from two murine models with intense muscle degeneration and regeneration.

**Methods:**

One murine lineage was a *Dmd*^*mdx*^ model of Duchenne muscular dystrophy (*n* = 6). In the other model, muscle damage was induced by electroporation in C57BL/6J wild-type mice, and analyzed after 5, 15, and 30 days post-electroporation (dpe; *n* = 15). Regeneration was evaluated based on the quantity of developmental myosin heavy chain (dMyHC)-positive fibers.

**Results:**

The frequency of fibers containing TA was higher in aged C57BL/6J (26 ± 8.3%) than in old dystrophic *Dmd*^*mdx*^ mice (2.4 ± 2%). Comparing the data from induced degeneration/regeneration in normal mice revealed a reduced proportion of TA-containing fibers after 5 and 30 dpe. Normal aged muscle was able to regenerate and form dMyHC+ fibers, mainly at 5 dpe (0.1 ± 0.1 vs. 16.5 ± 2.6%). However, there was no difference in force or resistance between normal and 30 dpe animals, except for the measurements by the Actimeter device, which showed the worst parameters in the second group.

**Discussion:**

Our results suggest that TA also forms in the *Dmd*^*mdx*^ muscle but in smaller amounts. The intense degeneration and regeneration of the old dystrophic model resulted in the generation of new muscle fibers with a lower quantity of TA. Data from electroporated wild-type mice support the idea that muscle regeneration leads to a reduction in the amount of TA. We suggest that TA accumulates in muscle fibers throughout physiological aging and that regeneration leads to the formation of new fibers without these structures. In addition, these new fibers do not confer functional benefits to the muscle.

## 1 Introduction

Tubular aggregates (TA) are skeletal muscle structures that arise from the progressive accumulation of sarcoplasmic reticulum (SR) proteins (1–5). Cytoplasmic aggregates of muscle fibers have been observed in both mice (6) and humans (3). These factors are relevant to aging and muscle disease processes. The pathophysiological mechanisms of many human neurodegenerative diseases, such as amyotrophic lateral sclerosis, Alzheimer’s disease, Parkinson’s disease, and Huntington’s disease, are intrinsically related to protein aggregation and inclusion body formation (7). Thus, there is a strong link between condensate-forming proteins and age-related diseases, such as neurodegeneration and cancer (8).

In humans, TA are found in diseased skeletal muscles such as tubular aggregate myopathy (TAM) (9), Andersen–Tawil syndrome (10), and limb-girdle myasthenia (11). Major differences between TAM and TA are associated with other myopathies. TA are prevalent in both slow-twitch (type I) and fast-twitch (type II) muscle fibers in TAM, whereas they are only present in type II fibers as a secondary effect in other myopathies. TAM affects mostly men, and in isolated TA, the proportion of women presenting with them is greater, although men are generally more affected (12).

TA have also been found in wild-type aged mice of different strains within muscle fibers, especially type II, but only in male specimens (5), which makes these structures age-, fiber type- and sex-specific (1).

The effects of muscle regeneration on TA have not yet been reported. This study aimed to compare the presence of TA in normal and dystrophic aged muscles and assess whether TA negatively influences muscle function after induced regeneration.

## 2 Animals and methods

### 2.1 Animals

**Figure 1:**
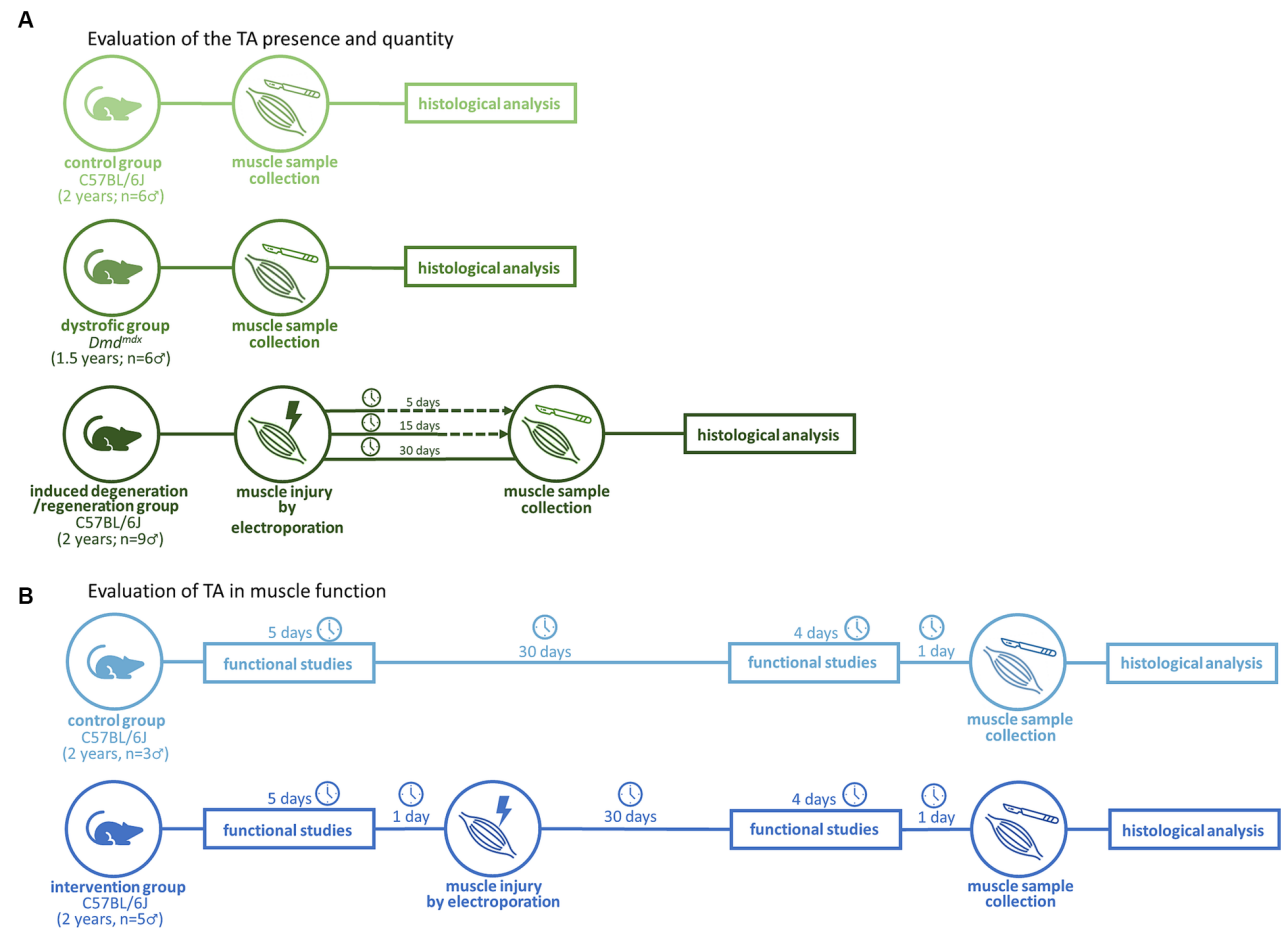
Experimental workflows for evaluation of the TA presence **(A)** and impact in muscle function **(B)**.

To evaluate the presence of TA, C57BL/6J (*n* = 15, 24 months of age) mice were subdivided into a control non-electroporated group (NE; *n* = 6) and three groups of animals submitted to electroporation in the calves and analyzed 5 days post-electroporation (dpe) (*n* = 3), 15 dpe (*n* = 3), and 30 dpe (*n* = 3). An additional group of naturally degenerated animals composed of *Dmd^mdx^* mice (model for Duchenne muscular dystrophy) (*n* = 6, 18–24 months of age) was also included (Figure 1A). All animals were males.

**Figure 2:**
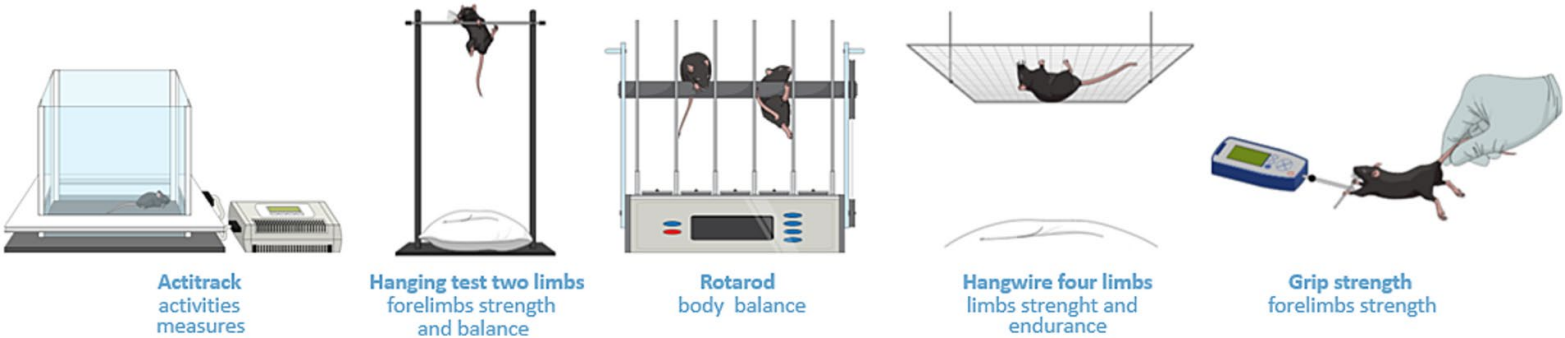
Methodologies for functional studies. Actitrack uses infrared sensors to record mouse movements, providing data that includes distance traveled and speed. The two-limb hanging test assesses forelimb strength and balance. The observer records the hanging time of the subject. The four-limb hanging test assesses strength and endurance by placing the mouse upside down, hanging from a grid. The rotarod test assesses body balance as the animal is positioned on a spinning metal cylinder. The grip strength test records the force produced by the animal while the researcher pulls its tail gently.

In the functional evaluation (Figure 1B), 2 years-old C57BL/6J (*n* = 8) mice were subdivided into the NE control group (*n* = 3) and electroporated group (*n* = 5), composed of animals subjected to electroporation of calves according to a previously described protocol (13). Functional evaluations included strength, resistance, and global activity (Figure 2). These tests were performed at 0 dpe time zero in both groups and at 30 dpe in the second group (Figure 1B).

### 2.2 Experimental procedures

#### 2.2.1 Global activity measurements

Actitrack equipment (Panlab, Barcelona, Spain; Figure 2) was used to measure the distance covered, speed, slow and fast movements, rest, and number of rearings. The velocity thresholds adopted were the equipment software standards (rest <2 cm/s, slow velocity between 2 and 5 cm/s, and fast velocity >5 cm/s). Each animal was monitored for 20 min with a 10 min break between animals. This assessment was repeated once daily for 4 days. On the fifth day, we performed the other tests as described below. For the second evaluation after 30 days, the Actitrack assessment was repeated for 3 days and the other tests were repeated on the fourth day.

#### 2.2.2 Assessment of strength and endurance

The grip strength, hanging test involving two and four limbs, and rotarod test (Figure 2) were performed based on the TREAT-NMD protocols (14), which consider the conversion of measures in newtons into grams, and divided these values by the respective weights.

#### 2.2.3 Sample collection

The animals were then anesthetized and euthanized. Gastrocnemius muscles were collected and stored in liquid nitrogen. Frozen tissues were cross-sectioned in a HM 505 E cryostat (Microm International GmbH, Walldorf, Germany) at 6–7 nm thickness for histological and immunofluorescence (IF) analyses.

#### 2.2.4 Staining processes

Hematoxylin and eosin (HE) staining was performed using a standard protocol aimed at assessing tissue integrity. Gomori trichrome (GT) staining was performed to verify the presence of TA in the muscle fibers, as previously described (15). Muscle fiber cytoplasm stained green, nuclei purple, and TA a purple-reddish color.

#### 2.2.5 IF of developmental myosin heavy chain

dMyHC is a protein present in immature fibers. It is a marker of muscular regeneration. Primary antibodies used for staining were a 1:30 dilution of mouse dMyHC-NCL, for developmental myosin staining (Novocastra NCL-MHCd, Leica Biosystems, Concord, ON, Canada) and a 1:100 dilution of rabbit anti-laminin (Z0097, Dako, San Diego, CA, United States) for extracellular matrix staining. The secondary antibodies used were a 1:200 dilution of Cy3 goat anti-mouse (A10521; Invitrogen, Carlsbad, CA, United States) and a 1:100 dilution of fluorescein isothiocyanate (FITC) goat anti-rabbit (F0511; Sigma-Aldrich, St. Louis, MO, United States). IF was visualized and analyzed using an Axio Imager.Z1 microscope (Zeiss, Jena, Germany).

#### 2.2.6 Morphometric analysis

We photographed entirely each gastrocnemius section stained by Gomori Trichrome. It allows us to correctly identify the myofibers contour and the TA presence, so we can count them individually, which was done in a global manner using the Fiji software (16). On average, 2,370 fibers were analyzed per animal. After that, we calculated the proportion of myofibers containing TA over total number of fibers counted in muscle section.

### 2.3 Statistical analysis

The statistical software GraphPad Prism v8.2.1 (GraphPad Software Inc., Boston, MA, United States) was used. The Shapiro–Wilk normality test was performed for each experimental group analyzed. If the distribution was normal, paired or unpaired student’s *t* parametric tests were used to compare two experimental groups, while one-way ANOVA with Dunnett’s correction was performed to compare more than two. If the distribution was not normal, the Mann–Whitney test (unpaired samples) or Wilcoxon test (paired samples; before vs. after) were used to compare two groups, while Kruskal–Wallis was perfomed to compare more than two groups. The confidence interval adopted for the statistics was 95%.

## 3 Results

### 3.1 TA are present, but in decreased quantity in dystrophic aged muscles

**Figure 3:**
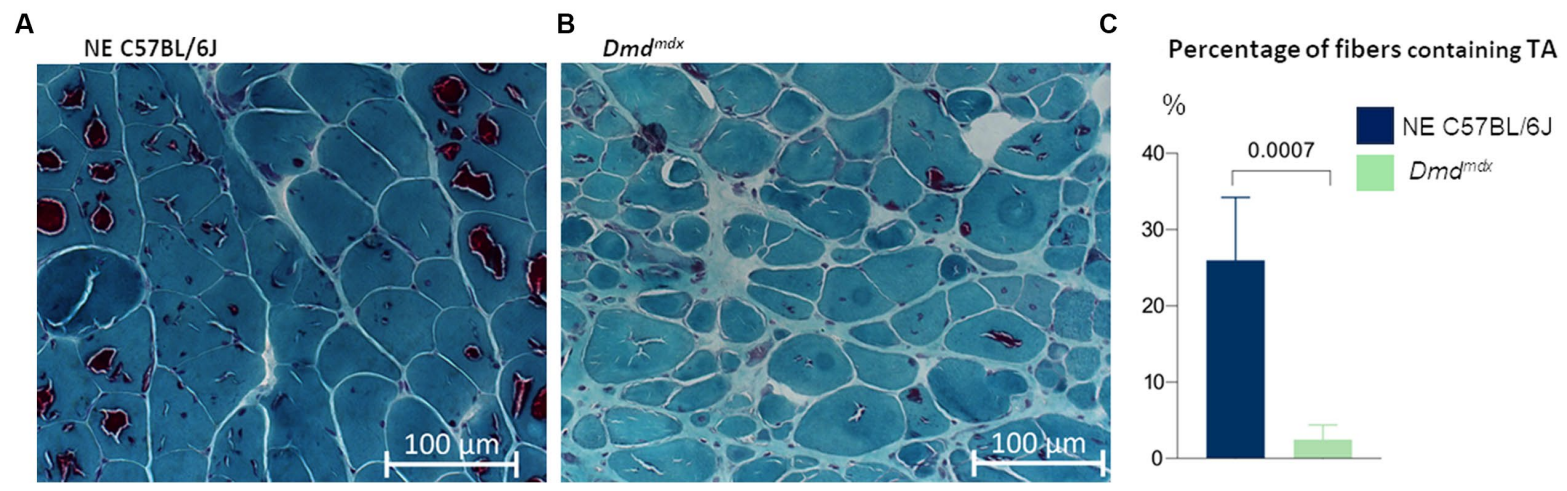
Gastrocnemius muscle from aged mice, stained with modified Gomori trichrome. TA are present in all experimental groups. There were more fibers containing TA in **(A)** C57BL/6J mice (*n*   =  6) than in **(B)** Dmd^mdx^ mice (*n*  = 6, *t*-test).

TA were detected in both aged normal and dystrophic *Dmd^mdx^* mice (Figure 3). However, more aggregates were evident in normal C57BL/6J mice than in *Dmd^mdx^* mice (26 ± 8.3% vs. 2.4 ± 2%, *p* = 0.0007, *t*-test; Figure 3C).

### 3.2 Induced degeneration causes decrease in number of TA after regeneration

**Figure 4:**
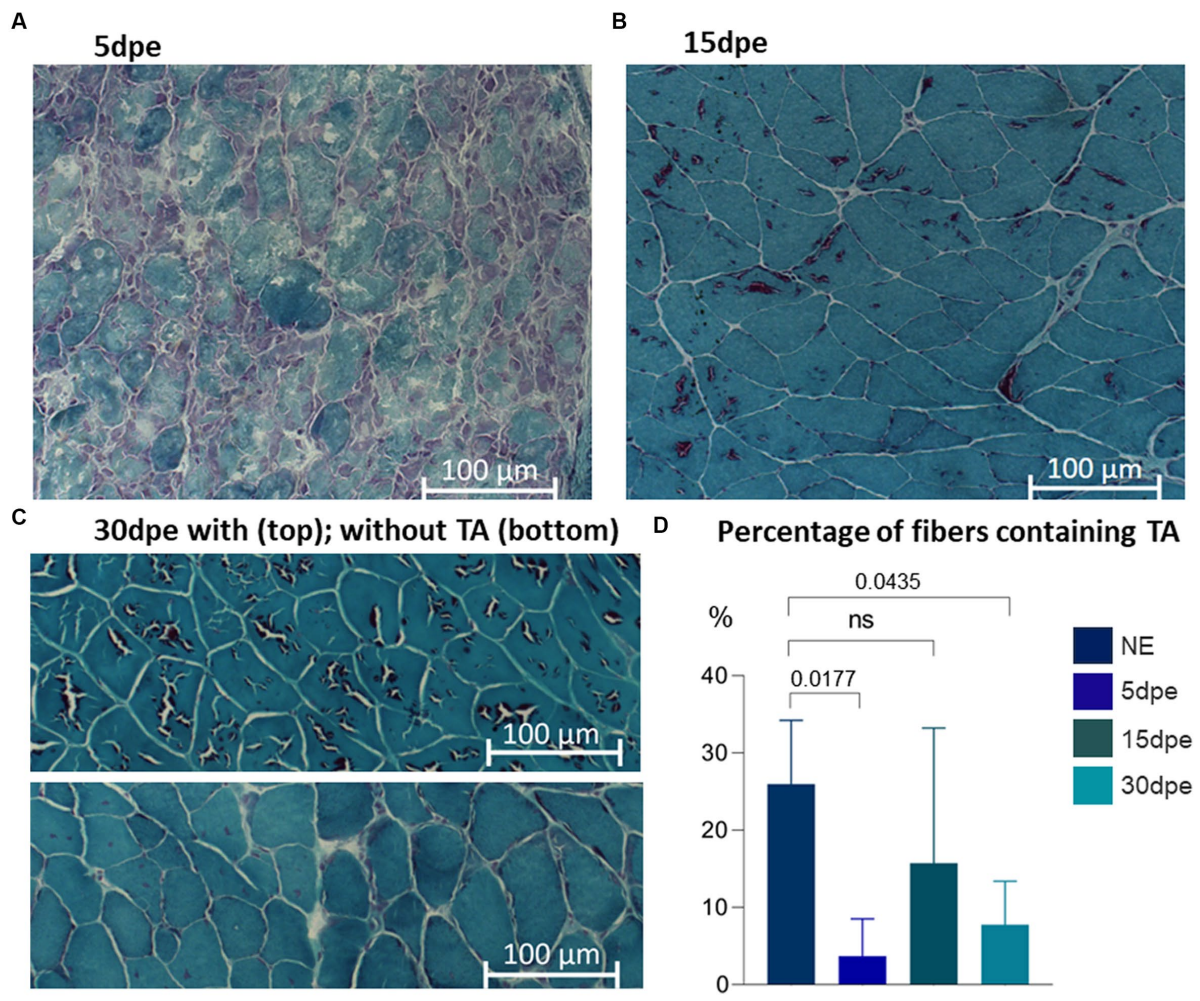
Gastrocnemius muscle from aged mice stained with modified Gomori trichrome. TA are present in all experimental groups. Intense degeneration is evident at **(A)** 5 dpe. After muscle regeneration, TA are present in some muscle fibers at **(B)** 15 and **(C)** 30 dpe. The proportion of fibers containing TA was less at 5 and 30 dpe (one-way ANOVA with Dunnett’s correction).

Comparison of the data from normal aged animals with induced degeneration-regeneration revealed a reduction in the proportion of fibers containing TA at all times post-electroporation. One-way ANOVA with Dunnett’s correction analysis showed that reductions were statistically significant after 5 dpe (3.7 ± 5%, *p* = 0.0177) and 30 dpe (7.7 ± 5.7%, *p* = 0.0435) (Figure 4). TA were different sizes and present as single or multiple units in each muscle fiber, and with or without internal content (staining inside the TA or only on the outer edge, respectively). In fibers with internalized nuclei (newly formed fibers), TA were not identified, although they were still present within nearby fibers.

### 3.3 Regeneration profile in old mice

**Figure 5:**
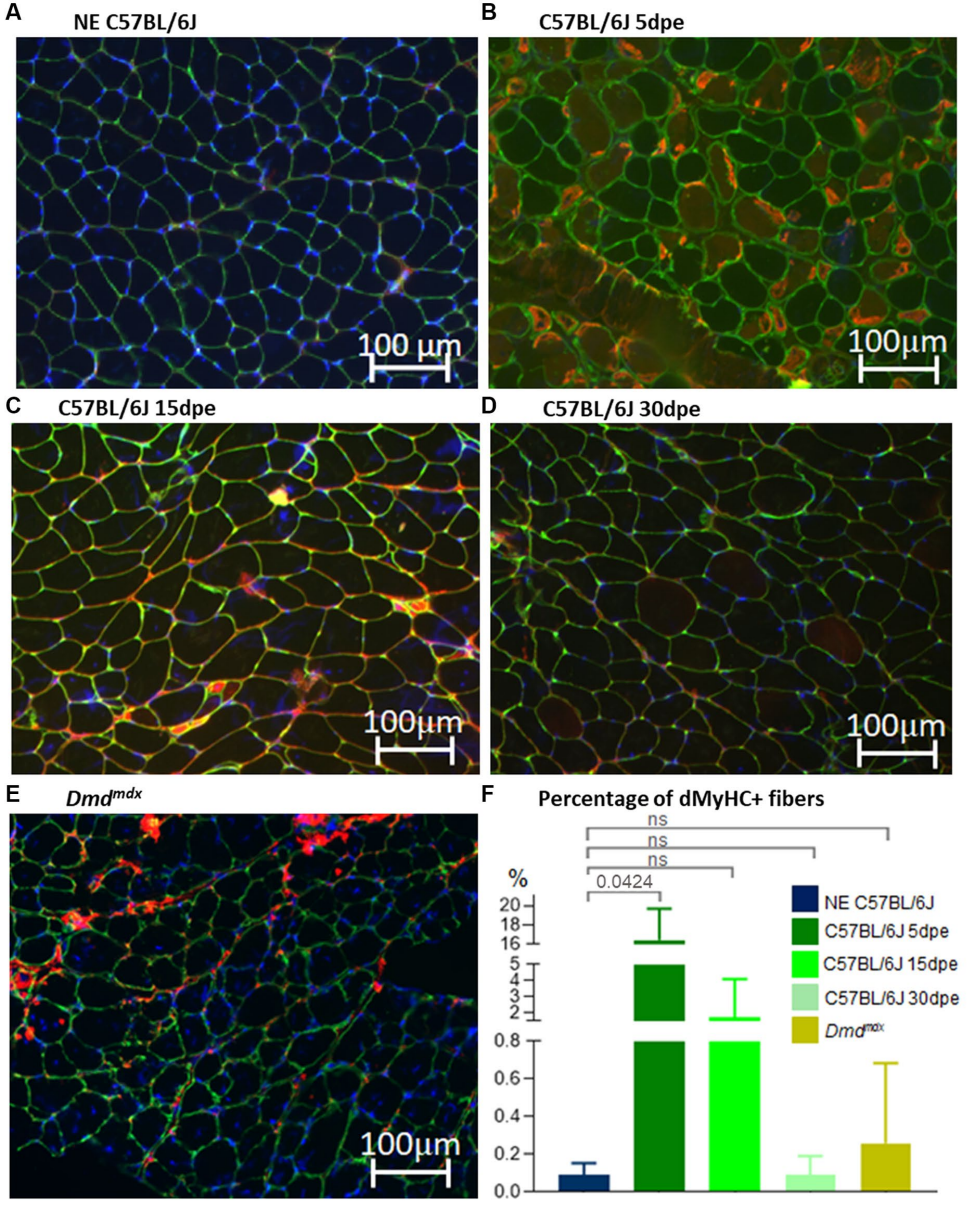
Gastrocnemius muscle sections of C57BL/6J before (NE, **A**) and after electroporation (5, 15, and 30 dpe, **B–D**) and in Dmd^mdx^ mice **(E)**. IF was performed using antibodies against dMyHC (red stain inside muscle fibers) and laminin (green stain). Nuclei are stained in blue by dapi (4’,6-diamidino-2-phenylindol). **(F)** Denotes the percentage of positive dMyHC fibers between experimental groups. The only significant difference was between NE C57BL/6J and 5 dpe mice (Kruskal–Wallis with Dunnett’s correction).

dMyHC-positive fibers were scarce in NE old C57BL/6J animals (0.1 ± 0.1%; Figure 5A) and *Dmd^mdx^* mice (0.3 ± 0.4%; Figure 5E), with no statistically significant difference between them (Mann–Whitney—*p* = 0.3864; Figure 5F). The lesion induced regeneration, with increase of dMyHC-positive fibers at 5 dpe (16.5 ± 2.6%; Figure 5B). The difference between NE and 5dpe C57BL/6J is statistically significant (Kruskal–Wallis with Dunnett’s correction—*p* = 0.0424; Figure 5F). From 15 dpe there was a reduction in dMyHC-positive fibers (1.8 ± 1.7%; Figure 5C). Thirty dpe animals (0.1 ± 0.1% - Figure 5D) presented a complete regeneration, with similar values to those of NE C57BL/6J. Comparison of 15 dpe and 30 dpe with NE animals showed no significant differences (Kruskal–Wallis with Dunnett’s correction—Figure 5F).

### 3.4 Decreased TA after muscle regeneration is not related to muscle functional improvement

**Figure 6:**
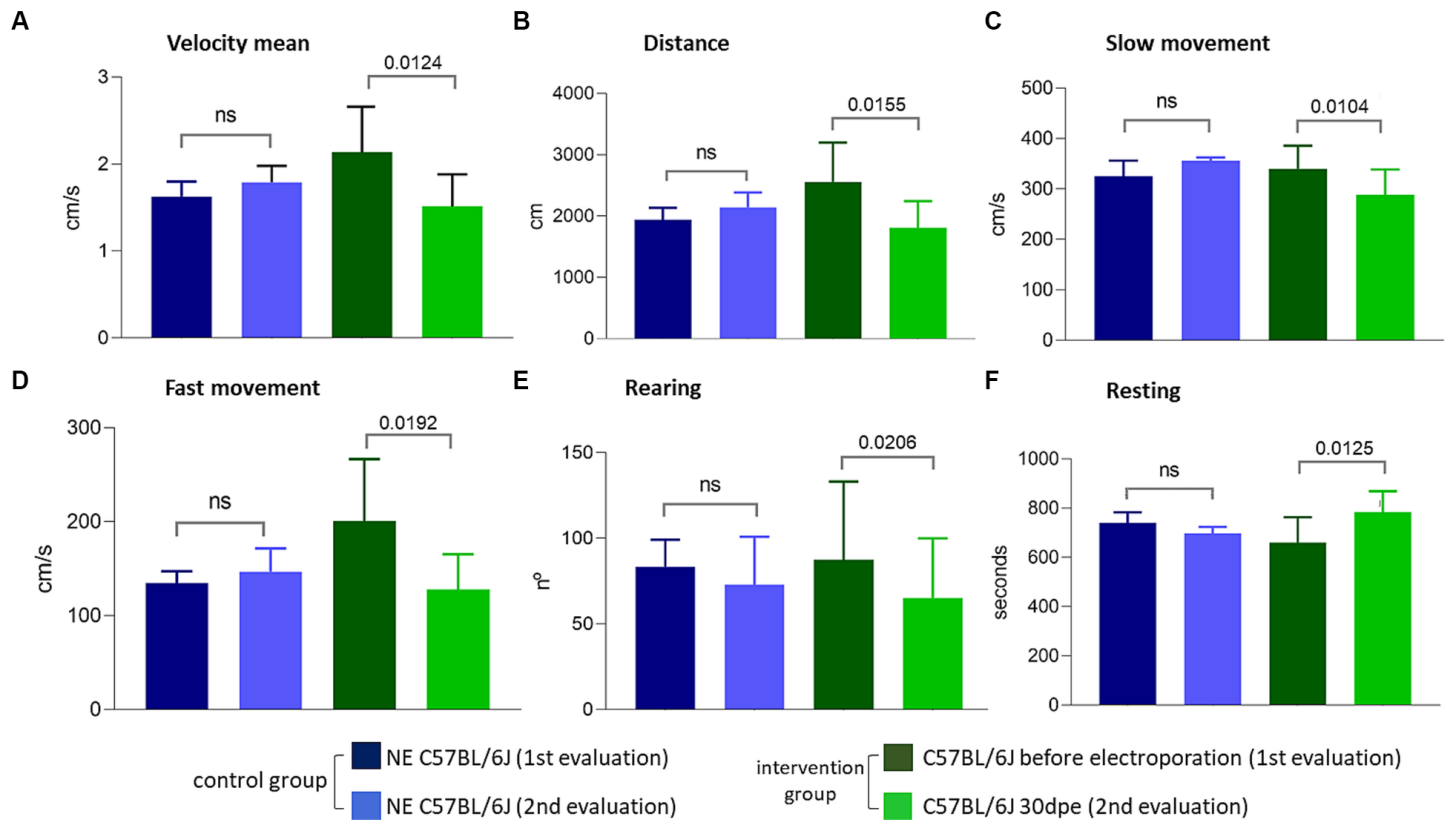
Evaluations performed using the Actitrack equipment. All comparisons **(A–F)** between the intervention group before electroporation and 30 dpe were statistically significant (paired *t*-test, *p* < 0.05), compatible with a worsening of the phenotype caused by electroporation.

**Figure 7:**
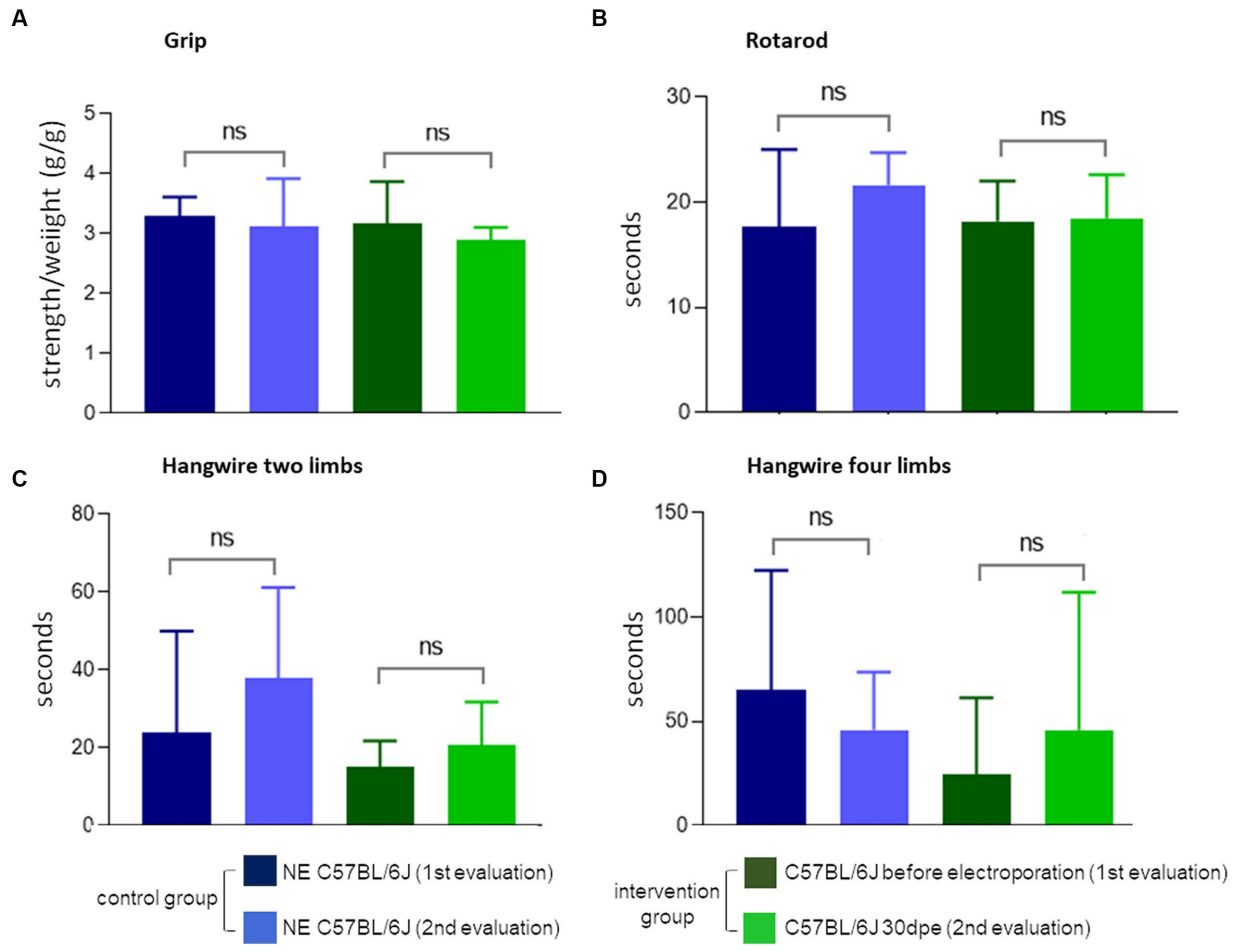
Force and balance measures. None of the four tests **(A–D)** showed the difference between normal NE C57Bl and 30 dpe post-injury animals (paired *t* or Wilcoxon tests).

The observation of less TA in regenerated muscles of aged normal mice after induced degeneration prompted the hypothesis that regeneration inducing the formation of new fibers with less TA could modify the functional capacity of the muscle. To verify this hypothesis, we repeated the experiment and induced degeneration in normal aged muscles and functionally evaluated these animals before and 30 days after injury and regeneration. Again, more fibers with TA were observed in NE animals (35.3 ± 12%) compared to electroporated animals at 30 dpe (23.8 ± 5.3%). The global activity measurements obtained using Actitrack equipment revealed statistically significant poorer performance of the electroporated animals compared to NE subjects. As shown in Figure 6, the indices of mean velocity (A), distance (B), slow movement (C), fast movement (D), and rearing (E) were significantly lower in the electroporated mice (30 dpe), whereas rest time (F) was higher in this group (paired *t*-tests). The animals were also evaluated using four tests for force and resistance: grip strength, rotarod, hanging with two limbs, and hanging with four limbs. No differences between the control and intervention groups were evident using paired *t* or Wilcoxon tests (Figure 7).

## 4 Discussion

In this study, we aimed to compare the capacity of the old muscle to regenerate, both in chronic model of degeneration, as observed in muscular dystrophies, as compared to acute degeneration, induced by muscle demage by electroporation. The number of positive dMyHCs was quantified to verify the efficiency of regeneration and formation of new muscle fibers in genetically degenerated dystrophin-deficient mice (*Dmd^mdx^*) and in normal muscles under induced degeneration. As observed in young normal mice subjected to electrical muscle injury (17, 18), new fibers positive for dMyHC clearly increased at 5 dpe. dMyHC-positive fibers were reduced in number at 15 dpe and reached normal values after 30 dpe. The findings indicate that aged normal muscle preserves its regenerative capacity and formation of new muscle fibers in a pattern similar to that observed in young normal animals. In contrast, old *Dmd^mdx^* mice showed a small increase in the number of positive new fibers. This result is different from the significantly greater prevalence of regenerated fibers observed in young *Dmd^mdx^* mice (17%) (17).

TA inside aged muscle fibers are more likely to form during muscular physiological senescence. TA are repositories of proteins that have accumulated for a long time (several months in mice or years in humans). Acute injury, such as electroporation, or chronic injury, such as dystrophic state, leads to a degeneration-regeneration process in which severely damaged fibers are removed from muscle and satellite cells are recruited. The cells differentiate into myoblasts and fuse with each other to form new fibers or with a lesioned fiber to repair the fiber (19). In the present study, new fibers lacked TA. In the first round of experiments, both degeneration-regeneration groups of mice (*Dmd^mdx^* and electroporated C57BL/6J) presented fewer fibers containing TA than the control group (NE C57BL/6J mice). These findings indicate that recently formed muscle fibers do not contain TA, possibly due to the short post-regeneration time.

The identification of TA is technically challenging. Previous studies using different staining protocols to visualize TA have generated inconsistent results (20). For example, TA stained positively with nicotinamide adenine dinucleotide dehydrogenase-tetrazolium reductase (NADH-TR) (21) but negatively in another study (6). Details are lacking concerning the mechanism of TA genesis, chemical content, and functional consequences. However, some relevant knowledge is known. For example, SR proteins such as calsequestrin (1, 2, 5), Serca1 (1, 2, 6) and Stim1 (22, 23) are found in TA. All these SR proteins are involved in calcium homeostasis (2, 23). Schiaffino et al. (5) proposed the origin of TA in the SR, and compared protein aggregation in muscle with that in neurodegenerative diseases, and discussed the rearrangement of endoplasmic reticulum membranes in non-muscle cells in a similar form to that observed in muscle. The authors suggested that hypoxia led to the formation of TA. Orai1 plasma membrane calcium channel protein (23) was also identified in muscle TA (2, 6, 22). Due to the protein composition of TA, it is possible that correct calcium homeostasis influences TA formation. It would be interesting to verify whether the abnormal elevation of intracellular calcium concentration in dystrophin-deficient muscle, which contributes to disease progression in Duchenne muscular dystrophy (24), could be related to a lower amount of TA in *Dmd^mdx^* dystrophic muscle.

TA have been demonstrated in different mouse strains (C57BL/6J, BALB/c, DBA/2, 129Sv, and 129Ola) since 5 months of age, with a subsequent increased prevalence within fibers to almost 80% in old gastrocnemius mice (18 months) (6). In addition, male mice fed a resveratrol-enriched diet from 12 to 18 months of age showed a reduction in TA and an improvement in capillarization per fiber (25). Despite the broad observation of TA in wild-type inbred mouse strains (6), TA has also been previously found in dystrophic mice (*Lama2^dy^* model for congenital merosin-deficient muscular dystrophy 1A) (26). TA were observed in heterozygous, but not homozygous, mice; in the latter, degeneration-regeneration is more intense. In the present study, the presence of TA was observed in another dystrophic model (*Dmd^mdx^*), specifically in old animals, but in a lesser quantity that observed in old NE C57BL/6J mice. These findings reinforce the hypothesis that TA requires time to accumulate in the sarcoplasm because the intense degeneration-regeneration process in dystrophic animals results in the formation of new fibers that do not have aggregate.

TA have also been observed in human muscle diseases. Funk et al. (12) reported TA in 103 myopathic patients with variable clinical findings and age of onset ranging from childhood to old age. TA presented in subsarcolemmal regions and stained positively for SERCA1 and SERCA2 in both fiber types. Interestingly, the TA stained positive for tau, a protein associated with neurodegenerative diseases. Jain et al. (9) reported four adult patients with TAM; TA appeared in type 1 fibers in two patients and in type 2 fibers in the other two. TA were positively stained by HE, GT, and NADH-TR but were not stained by SDH and Cox stains. All patients presented with muscle weakness. Vivekanandam et al. (10) reported TA (as subsarcolemmal structures) in two of five biopsies of Andersen–Tawil syndrome, which affects most patients with episodic muscle weakness. Limb-girdle myasthenia is another example of a disease in which TA could be present, with a reported patient with clinical signs at 5 years of age. TA in his muscle was revealed at 23 years of age by biopsy (11). In all these reported cases (9–12), TA did not seem to be the cause of the disease. The eventual contribution of TA to pathophysiology or even to the worsening of clinical signs has not yet been investigated.

The functional assessments in the present study measured the physical aptitude of aged mice with more or less TA within muscle fibers. The aim was to see if the absence of TA in new fibers could affect muscle function. However, we did not consider that this *in vivo* experimental design could be influenced by other changes in the tissue caused by the injury, such as loss of muscle mass and consequent loss of strength, fiber type change, and diminished motor units (motor neuron and the myofibers activated by it) (27, 28). The experimental design could not assess the presence of TA as the only variable among the analyzed groups. Moreover, the absence of differences in these tests may have been due to the limited number of animals used.

A possible strategy for TA reduction is exercise (22). Exercise improves health, especially for the elderly population. This has global importance, given that it is expected to be 1 billion people ≥60 years of age worldwide by 2030 (29). During aging, progressively increasing cellular death and malfunction result in sarcopenia and osteoporosis, featuring loss of muscle and bone mass, respectively (30). Locomotor skills can be impaired, reducing the autonomy of the older population in their daily activities. A better understanding of age-related dysfunctions, such as the aggregation of intracellular structures, would help in tracing targets for therapies and other strategies to improve healthier aging.

## 5 Conclusion

TA form over time, appears in aging normal murine muscles. TA reduction in injured conditions may be due to the degeneration-regeneration process in muscles, with loss of damaged muscle fibers and formation of new fibers that do not present protein aggregation. These new regenerated fibers do not improve the functiona capacity of the aged muscle.

## Data availability statement

The raw data supporting the conclusions of this article will be made available by the authors, without undue reservation.

## Ethics statement

The animal study was approved by Comissão de Ética no Uso de Animais em Pesquisa, Instituto de Biociencias, Universidade de São Paulo. The study was conducted in accordance with the local legislation and institutional requirements.

## Author contributions

FV: Conceptualization, Data curation, Formal analysis, Validation, Writing – original draft, Writing – review & editing, Investigation, Methodology. AJ: Data curation, Investigation, Writing – review & editing. BS: Writing – review & editing, Methodology. IA: Methodology, Writing – review & editing. LC: Writing – review & editing. LF: Writing – review & editing, Methodology. LS: Methodology, Writing – review & editing. NS: Methodology, Writing – review & editing. MF: Writing – review & editing, Formal analysis. MV: Formal analysis, Writing – review & editing, Conceptualization, Data curation, Funding acquisition, Project administration, Supervision, Validation, Writing – original draft.
